# Misinformation about the recent hantavirus outbreak on a cruise ship

**DOI:** 10.1080/22221751.2026.2695532

**Published:** 2026-08-10

**Authors:** Yi-Wei Tang

**Affiliations:** School of Public Health, Chongqing Medical University, Chongqing, People’s Republic of China

**Keywords:** Andes virus, hantavirus, cruise ship outbreak, misinformation, risk communication

## Abstract

The recent Andes virus (ANDV) outbreak associated with the cruise ship MV Hondius generated substantial misinformation on social media. This editorial addresses six prominent myths concerning pandemic potential, airborne transmission through ventilation systems, unproven treatments, risks associated with cruise travel, influenza-like transmissibility, and claims that the outbreak was fabricated. Current evidence indicates that, although ANDV can rarely spread between people following close and prolonged contact, its transmission differs fundamentally from that of highly contagious respiratory viruses. The outbreak highlights the need for scientists, public health authorities, healthcare professionals, and the media to communicate evidence rapidly and transparently. Effective responses must control not only emerging infections but also the accompanying spread of misinformation.

The power of social media in our time, not surprisingly, generated lots of myths circulating around the recent Andes hantavirus (ANDV) outbreak linked to a sailing cruise ship, MV Hondius, in the South Atlantic Ocean [1–4].

Even though public health authorities continue to emphasize that the overall risk of hantavirus to the public is low, misinformation narratives have already circulated around the recent ANDV cases reported. Being someone who worked my entire life in research, clinical service, and academic publishing focused on emerging infections, including hantavirus [5, 6], I feel obligated to provide a professional response to the following six myths for the benefit of the general public.

“**This is the next COVID pandemic.**”

Experts and WHO officials have repeatedly said this is not comparable to COVID-19 in transmissibility. ANDV is transmitted person-to-person, but only rarely and usually through close, prolonged contact with symptomatic individuals, and is not spread via air [6, 7].

“**The virus is spreading easily through cruise ship ventilation systems**.”

Social media speculation suggested widespread airborne transmission through ship air conditioning HVAC. Investigators have not confirmed this, and current evidence still points mainly towards rodent exposure and limited close-contact transmission [3,7].

“**Ivermectin or other unproven drugs can prevent/treat hantavirus**.”

Epidemiologists reported that some influencers rapidly promoted ivermectin and other unsupported treatments online, echoing patterns seen during COVID-19 misinformation campaigns [8]. There is currently no established antiviral cure specifically proven for ANDV infection (https://www.politifact.com/article/2026/may/11/hantavirus-outbreak-treatments-ivermectin/?utm_source=chatgpt.com).

“**Cruise ships are inherently breeding grounds for any virus**.”

Commentary pieces and social media amplified the idea that cruises inevitably cause major outbreaks. While cruise ships can facilitate transmission because of close quarters, especially for these highly contagious viruses such as measles and COVID-19, experts note that this outbreak appears unusual because ANDV outbreaks are themselves rare [9, 10].

“**Hantavirus spreads like influenza or measles**.”

Public health agencies have stressed that hantaviruses are fundamentally different. Most hantaviruses are not efficiently transmitted between humans at all; ANDV is one of the few exceptions, and even then, transmission person-to-person is limited [7, 8].

As we have seen in recent years, some misinformation is created based on conspiracy theories:

“**There was no real outbreak; it was fabricated**.”

Some online posts claimed the cruise outbreak itself was fake or exaggerated. However, WHO, CDC, Reuters, and multiple governments confirmed the cases, quarantines, and deaths (https://www.reuters.com/business/healthcare-pharmaceuticals/who-revises-hantavirus-cases-lower-after-us-passenger-tests-negative-2026-05-15/?utm_source=chatgpt.com).

With enhanced diagnostic tests and superfast online communication tools worldwide, we will hear outbreaks of human infections more often than before; the scientific community needs to share the true nature and details of these outbreaks so our normal lives will not be unnecessarily affected [11, 12].

Be alert, but do not panic. Perhaps the greatest lesson from this outbreak is that misinformation may travel faster than the virus itself. Public health authorities, scientists, healthcare professionals, and the media must work together to ensure that facts – not fear – shape public understanding and response.
